# The Consequentialist Scale: Translation and empirical investigation in a Greek sample

**DOI:** 10.1016/j.heliyon.2023.e18386

**Published:** 2023-07-17

**Authors:** George Kosteletos, Ioanna Zioga, Evangelos D. Protopapadakis, Andrie G. Panayiotou, Konstantinos Kontoangelos, Charalabos Papageorgiou

**Affiliations:** a University Mental Health, Neurosciences and Precision Medicine Research Institute “COSTAS STEFANIS” (UMHRI), Athens, Greece; b Applied Philosophy Research Laboratory, National and Kapodistrian University of Athens, Athens, Greece; c Donders Institute for Brain, Cognition and Behaviour, Radboud University, Nijmegen, the Netherlands; d First Department of Psychiatry, National and Kapodistrian University of Athens Medical School, Eginition Hospital, Athens, Greece; e Open University of Cyprus, Nicosia, Cyprus; f Cyprus International Institute for Environmental and Public Health, School of Health Sciences, Cyprus University of Technology, Limassol, Cyprus

**Keywords:** The consequentialist scale, Consequentialism, Deontology, Moral ideologies, Exploratory factor analysis, Age effect

## Abstract

The Consequentialist Scale (Robinson, 2012) [89] assesses the endorsement of consequentialist and deontological moral beliefs. This study empirically investigated the application of the Greek translation of the Consequentialist Scale in a sample of native Greek speakers. Specifically, 415 native Greek speakers completed the questionnaire. To uncover the underlying structure of the 10 items in the Consequentialist Scale, an Exploratory Factor Analysis (EFA) was conducted. The results revealed a three-factor solution, where the deontology factor exhibited the same structure as the original work by Robinson (2012) [89], while the original consequentialism factor split into two separate factors. Significant Pearson's *r* correlations were observed between age and responses to the Consequentialist Scale. Separate EFAs were conducted for two age groups based on a medial split: younger (36 years old or less) and older (more than 36 years old). Interestingly, the younger group exhibited a two-factor solution with the same structure as the original work, while the older group showed a three-factor solution. A hierarchical k-means cluster analysis revealed that the cluster of participants who scored higher in deontology compared to consequentialism primarily consisted of older participants, whereas the two other clusters comprised of younger participants exhibited the reverse pattern. Neither gender nor previous experience with philosophy significantly affected scores on the Consequentialist Scale. Overall, our study provides evidence that the Consequentialist Scale is suitable for use in the Greek population.

## Highlights

• The first empirical investigation of the Consequentialist Scale in a Greek sample.
• An Exploratory Factor Analysis was conducted, revealing a three-factor solution.
• Additional investigation revealed a significant age effect.
• A potential effect of culture was also considered.

## 1 Introduction

### 1.1 Deontology vs. consequentialism

Morality is a prominent characteristic of humans, and moral judgment constitutes a significant and distinct aspect of human decision-making. Philosophers have proposed several frameworks of morality, such as virtue ethics, contractualism, care ethics, W.D. Ross' “prima facie duties”, and others, which may guide lay people's moral reasoning. Among these frameworks, deontology, and consequentialism have received the most attention in empirical psychological research [1,2,3], although, in the past two decades, moral psychologists have also focused on other frameworks, such as the Moral Foundations Theory [4,5].

Deontologists argue that choices, actions, and intentions should be morally assessed based on their conformity with moral laws and principles, which admit no exceptions. Moral laws and rules are considered to be universal and intrinsically valuable, accepted and followed for their own content. In the philosophical tradition of Immanuel Kant [6], actions must align with one's own maxims, which are determined solely by reason and free from external constraints, thereby exhibiting autonomy [7,8]. On the other hand, consequentialists maintain that moral assessment should be based solely on the consequences of choices, actions, or intentions [9,10,3] and utilitarianism is considered the “paradigm case of consequentialism” [11].

### 1.2 Moral ideology questionnaires

The term “moral ideologies” (or “ethical ideologies”) refers to people's theoretical predispositions regarding the most appropriate way of analyzing a morally-laden case and reaching a final moral judgment. Despite their difficulties and drawbacks, deontology and consequentialism remain fundamental theoretical frameworks of moral thinking. Over the years, several questionnaires have been developed to identify individuals' propensities towards deontological or consequentialist moral thinking. For instance, Brandy and Wheeler [12] developed the Measure of Ethical Viewpoints (MEV), and Brandy [13] developed the Survey of Ethical Theoretic Aptitudes (SETA). However, these questionnaires have certain limitations, such as inconclusive subscale correlations in the case of MEV and forced-choice answers without opposite anchors in SETA. The Managerial Value Profile (MVP) [14] categorizes respondents into three ethical frameworks: social justice, deontology, and utilitarianism. However, it fails to account for the possibility that individuals might employ a combination of these frameworks [15]. Another relevant questionnaire is the Multidimensional Ethics Scale (MES) [16,17]. MES presents respondents with scenarios that conclude with certain actions taken by the protagonists. Participants are then asked to judge these actions using scale-based questions intended to reveal propensities towards five ethical foundations: justice, relativism, egoism, utilitarianism, and deontology [16]. However, the MES also has some drawbacks, such as high cross-scale correlations (see [18]).

One of the most widely-used questionnaires in the field of moral thinking is the Ethics Position Questionnaire (EPQ) [19]. The EPQ is based on the theoretical framework of the Ethics Position typology or “taxonomy of personal ethical ideologies'' [19,3]. This taxonomy recognizes the distinction between idealism and relativism, which is further refined into four sub-categories: situationism, absolutism, subjectivism, and exceptionism. These sub-categories emerge from the combination of high/low idealism and high/low relativism scores. Thus, unlike the MVP, the EPQ acknowledges the possibility that individuals may employ a combination of different ethical frameworks. However, the EPQ only has a partial and indirect link to the deontological and consequentialist frameworks of moral ideologies (for the relation between Forsyth's Ethics Position typology and the deontology/consequentialism framework, see [20]).

Two more recent questionnaires have been developed specifically for detecting propensity towards deontological or consequentialist moral thinking: the Consequentialist Scale [21] and the Ethical Standards of Judgment Questionnaire [15]. The Consequentialist Scale consists of 10 items that are rated on a 5-point Likert scale. Half of the items assess the endorsement of deontological beliefs, while the other half assess the endorsement of utilitarian beliefs. Similarly, the Ethical Standards of Judgment Questionnaire also includes a consequentialism and a deontology sub-scale, with 6 items rated on a 5-point Likert scale. Both questionnaires are concise, straightforward, and user-friendly, with the Consequentialist Scale having been slightly more widely utilized in research compared to the Ethical Standards of Judgment Questionnaire (for instance, see [22,23,24,[25], [26], [27]]).

### 1.3 The effect of culture

While there is an abundance of questionnaires available for investigating moral ideologies in the Western—particularly Anglo-Saxon—world, the generalizability of their results across populations belonging to different cultural contexts is being questioned. Numerous studies have examined the effect of culture on moral ideologies, including cross-cultural studies [28,29,30,31,32], within-culture studies [33,34,35,36], validation studies [37,38], and cross-cultural validation studies [39]. Empirical data suggest cultural variations in moral ideologies (for a meta-analysis of such an effect on the Ethics Positions typology see [40]). Several cultural characteristics like relational mobility [41] and elements of Hostede's typology [42] have been empirically linked with such variations [43,44,45,46,47,48].

However, to the best of our knowledge, there are no published cross-cultural studies available specifically on the Consequentialist Scale (CS). Furthermore, the questionnaire has not been validated in cultural contexts other than the American one. Considering the CS's brevity, practicality, and satisfactory construct validity in U.S. populations, it could serve as a valuable tool for studying moral ideologies in various cultural contexts. Here, we examined the factor structure of the CS when applied to a sample of native Greek speakers. Currently, there is a lack of research instruments available for studying moral cognition in the Greek cultural context, resulting in a scarcity of empirical studies on moral reasoning among Greeks and Greek-Cypriots. Thus, our examination represents an initial but crucial step toward the development of a toolbox of reliable research instruments for investigating moral cognition in this population.

### 1.4 The effect of age

Extending the work of Piaget, Kohlberg proposed a theory of moral development and was probably the first to empirically study the changes in people's moral reasoning through their lifespan in a systematic way [49,50]. This research direction was then followed by other researchers (e.g. Refs. [51,52]). Several studies have reported an age effect on moral cognition, specifically in the domains of moral ideologies and moral judgment. Concerning moral ideologies, the majority of studies indicate that older individuals tend to exhibit a preference for deontology and idealism, while younger individuals lean more toward utilitarianism and relativism [53,54,55,56,57,58,59,60,61,62]. Although there are a few studies that report opposite findings (e.g., see [63]). This age effect has been observed across cultures [54,59]. With regard to moral judgment, a similar age effect has been observed, with older individuals showing a greater inclination towards deontological and idealistic modes of judgment (or a reduced preference for utilitarian judgments), while younger individuals show a tendency towards more utilitarian and relativistic modes of judgment [64,65,66,58,67]. This age effect has also been found across cultures [64,67].

Different explanations have been proposed to account for the effect of age on moral ideologies and moral judgment. Some researchers argue that this age effect can be attributed to older individuals’ tendency to display more negative affective reactions [58] and to generally exhibit greater emotional and empathetic involvement in interpersonal contexts [64] (see also [68]). This explanation is further supported by the observation that older adults differ from younger adults in their inclination towards deontological decisions primarily when confronted with immediately compelling or intuitive dilemmas which typically elicit automatic, unreflective responses and evoke immediate emotional reactions [69].

In general, these interpretations of the effect of age on moral reasoning are consistent with empirical findings that demonstrate higher emotional responses among older individuals compared to younger individuals in situations eliciting sadness, such as personal or social loss [70,71,72]. These findings, along with studies showing that Idealism is positively associated with both cognitive and affective aspects of empathy [73], provide a solid explanation for the age-related differences in moral reasoning.

Another possible explanation for the age effect on moral reasoning can be derived from studies highlighting that, as individuals age, the ability to regulate and manage their emotional states becomes more crucial [74] (see also [75,76]). Choosing a deontological framework of moral reasoning may serve as a strategy to avoid experiencing further negative emotions, particularly those associated with utilitarian sacrificial choices [58]. Additionally, research drawing from terror management theory has provided insights into the relationship between mortality salience and utilitarian judgments. When individuals are primed with thoughts of death, they exhibit a reduced preference for utilitarian judgments in moral dilemmas [77]. In times of crisis, such as the current Covid-19 pandemic, older people provide fewer utilitarian responses to moral dilemmas involving personal rights [78].

The aforementioned studies demonstrate that age has an impact on the content of moral reasoning. However, empirical findings show that age can also impact the strictness and consistency of moral reasoning. Findings consistently show that older individuals across cultures [79] exhibit greater consistency, philosophical reflection, and stricter moral thinking with more developed and entrenched moral beliefs [53,80,81,56,58,82,60]. On the other hand, younger people are less consistent, more selfish, and more relativistic [55,56,57]. This relates back to older people's tendency towards negative affectivity, greater emotional engagement, and empathy [64,58].

Given the substantial body of research indicating an age effect on moral ideologies, we investigated whether age would affect the replication of the factors identified in the Consequentialist Scale (CS) within our sample of participants.

### 1.5 Aim of the present study

The current study investigated the factor structure of the Consequentialist Scale (CS) in a representative sample of native Greek speakers and obtained mean scores for moral ideologies in the Greek cultural context. Moreover, we explored the potential effect of age on moral ideologies and its impact on the replication of the original CS factors. To the best of our knowledge, this is the first comprehensive investigation of a moral ideologies scale in the Greek context, filling a significant research gap.

## 2 Methods

### 2.1 Participants

Four hundred and fifteen adults (133 female) aged between 18 and 67 years old (mean ± s.d. age of 35.5 ± 12.8 years) voluntarily participated in an anonymous online study consisting of the Consequentialist Scale and a series of demographic questions. All participants were native Greek speakers, with 366 having Greek nationality and 49 having Greek-Cypriot nationality. Participants were provided with detailed information about the procedure and gave online consent prior to participation. All procedures employed conformed to the Declaration of Helsinki. The study was approved by the Local Ethics Committee of the First Department of Psychiatry (National and Kapodistrian University of Athens Medical School, Eginition Hospital, Athens, Greece) and the National Bioethics Committee of Cyprus. The study was performed under the collaboration of the First Department of Psychiatry (National and Kapodistrian University of Athens Medical School, Eginition Hospital, Athens, Greece), the University Mental Health, Neurosciences and Precision Medicine Research Institute “Costas Stefanis”-UMHRI (National and Kapodistrian University of Athens, Greece), the Applied Philosophy Research Lab (National and Kapodistrian University of Athens, Greece) and the Cyprus International Institute for Environmental and Public Health (School of Health Sciences, Cyprus University of Technology, Limassol, Cyprus).

### 2.2 Materials and procedure

We tested the perception of the Consequentialist scale in a Greek sample. The scale, developed by Jeffrey Robinson [21], assesses the propensity for deontological (rule-based) vs. consequentialist (utilitarian) ethical beliefs. It consists of ten items, with five items assessing deontological beliefs and five assessing utilitarian beliefs. The scale begins with the following instructions: “Please indicate if you agree or disagree with the following items. Each represents a commonly held opinion and there are no right or wrong answers. We are interested in your reaction to such matters of opinion. Rate your reaction to each statement by choosing a number from 1 to 5 where: 1 = completely disagree; 2 = disagree; 3 = neither agree or disagree; 4 = agree; 5 = completely agree”. The items are presented in Table 1. The scale has been previously used to examine the relationship between utilitarian/deontological moral reasoning and attachment style [25]. In a pre-test with 1205 American participants, the scale demonstrated good internal reliability for both the deontological subscale (α = 0.74) and the utilitarian subscale (α = 0.83). Furthermore, the original construction and validation study conducted in a U.S. sample reported internal reliabilities of 0.64 and 0.80, respectively [21]. The scale has also been utilized in U.S. samples to study the relationship between an actor's level of exertion and observers' moral judgments regarding consequentialist or deontological decisions [26]. Additionally, it has been used in a study examining the potential relationship between disgust and the propensity for deontological/consequentialist beliefs in an American sample [27], as well as a study exploring the role of deontological and consequentialist reasoning in predicting online pro-social value endorsing crowdlending behavior in an Indonesian sample [33]. More recently, a shortened version of the CS with 4 items was employed in a Czech study examining preferred central (governmental/organizational) decisions in the face of a moral hazard [83]. However, to ensure the applicability of the CS in the Greek cultural context, it is necessary to investigate its factor structure in a Greek sample that is as representative as possible of the general Greek population. Therefore, the present study involved the translation of the Consequentialist Scale into Greek and a factorial analysis in the responses obtained from the aforementioned sample of participants.

**Table 1:** The items of the Consequentialist Scale [21].

Item	Statement
1	Some rules should never be broken.
2	It is never morally justified to cause someone harm.
3	If an action is a violation of societies most basic rules it should not be committed; even if it will result in a large amount of good.
4	Some aspects of humanity are sacred and should never be violated no matter the possible gain.
5	Some rules and laws are universal and are binding no matter the circumstances you find yourself in.
6	Rules and laws are irrelevant; whether an action produces happiness is all that matters when deciding how to act.
7	Rules and laws should only be followed when they maximize happiness.
8	If rules and laws do not maximize happiness for people they should be ignored.
9	The only moral principle that needs to be followed is that one must maximize happiness.
10	People that fail to maximize happiness are doing something morally wrong.

The translation of the Consequentialist Scale into the Greek language followed appropriate translation/back-translation protocols [84,85]. Two professional translators, both native Greek speakers with fluency in English, independently translated the original English questionnaire into Greek. This resulted in two Greek translations of the Consequentialist Scale, which were then combined to create a preliminary Greek translation. The preliminary Greek translation was subsequently translated back into English by a third professional translator, who was also a native Greek speaker with fluency in English. The resulting English translation and the original English version of the questionnaire were compared by the three translators and four of the authors (Kosteletos, Zioga, Protopapadakis, and Papageorgiou). This aimed to identify any substantial differences between the two English translations and make final adjustments to the Greek version accordingly. With these adjustments, the final Greek version of the Consequentialist Scale was produced. The Greek translation of the questionnaire is included in full in the Supplementary Material.

With regards to the demographic section of the questionnaire used in this study, in addition to the standard demographic questions about age, gender, and profession, we included two additional questions to explore potential effects of previous experience with philosophy. These questions were as follows: 1) *Have you studied anything related to Philosophy?* And 2) *Do you have any -even amateur level-engagement related to Philosophy (*e.g.*, reading of philosophical texts, audition of philosophical lectures/seminars, etc.)?*

We used Google Forms (www.google.com/forms/) to develop an online version of our experiment. The survey was distributed through our networks to various departments of the National and Kapodistrian University of Athens, Greece, and the Applied Philosophy Research Lab, as well as via social networks in Greece and networks related to the Cyprus International Institute for Environmental and Public Health, in Cyprus. Prior to the questionnaire, participants were presented with information about the study, as well as on the anonymity and confidentiality of their data and their ability to withdraw at any time.

### 2.3 Statistical analysis

#### 2.3.1 Exploratory factor analysis (EFA) of the consequentialist scale items

To examine the relationship between the items of the Consequentialist scale as arose from the responses of our Greek sample, we performed an exploratory factor analysis (EFA). EFA was suitable for our investigation as we did not want to bias the results with prior expectations with regard to the underlying factors. Specifically, EFA provides information on factor loadings for every item and factor, shedding light onto how the items are correlated to form conceptual dimensions. We first performed an EFA on the whole sample. After conducting Pearson's *r* correlations between responses in the items of the Consequentialist scale and age, we conducted separate EFAs for two age groups based on a medial split: the younger group (less or equal than 36 years old; *N* = 215, *M* ± *SD* 24.9 *±* 5.4) and an older group (more than 36 years old; *N* = 198, *M* ± *SD* 47.0 *±* 7.5). Reliability estimates of the factor scores were obtained by calculating the Cronbach's *α* reliability coefficients including the items defining each of the factors.

#### 2.3.2 Hierarchical k-means clustering of participants

To explore whether there were participants with similar patterns of responses and to identify and split them into separate clusters, we input their continuous consequentialism and deontology scores in a hierarchical k-means clustering analysis. We thus performed this analysis to split the participants into several groups (clusters) in a way that the participants in the same group are as similar as possible and the participants in different groups are as dissimilar as possible, with regards to their C and S scores. Specifically, the scores were computed as the sum of the items related to each factor, z-scored, and converted to STEN scores, for simplicity and interpretability purposes. STEN scores are computed by multiplying the z-score with the standard deviation and adding the mean. To identify clusters of participants with a similar profile, a hybrid hierarchical k-means clustering procedure was followed. Specifically, we first performed a hierarchical cluster analysis on the scores of each participant to determine the appropriate number of clusters. Euclidean distances were used as the distance metric and Ward's linkage as the clustering method. A scree plot revealed that the optimal number of clusters was three. Subsequently, a non-hierarchical k-means clustering was performed using the set of cluster centers as initial cluster centers, as derived from the aforementioned hierarchical procedure. This algorithm improved the initial clustering by minimizing the Euclidean distance between each data point and the nearest centroid. Once this process is completed for all k number of centroids, it is iterated to identify the best centroid positions. Cluster 1 (*N* = 140) showed a medium Deontology score (D score) and the highest Consequentialism score (C score), cluster 2 (*N* = 122) showed the lowest D score and a medium C score, while cluster 3 (*N* = 153) exhibited an opposite pattern, with the highest D score and the lowest C score. A 2 (factor: D, C) x 3 (cluster: 1, 2, 3) mixed ANOVA on the scores was conducted to confirm the presence of distinct clusters.

#### 2.3.3 Relationship between age, gender, experience with philosophy, and responses in the consequentialist scale

First, we investigated whether age was related to the different clusters of participants by performing a one-way ANOVA with three levels (clusters: 1, 2, 3). Furthermore, chi-square tests were conducted to investigate potential associations between gender (male, female), studies related to philosophy, engagement with philosophy, and cluster membership.

## 3 Results

### 3.1 Exploratory factor analysis

#### 3.1.1 Data assessment

We first assessed the suitability of our data for performing exploratory factor analysis (EFA). Specifically, we tested the factorability of the correlation matrix, by inspecting the correlation matrix of the 10 C S items (see Fig. S1 in Supplementary material). None of the items were too highly correlated (*r* < 0.8). To measure the proportion of variance among items that might be common variance, we calculated the Kaiser-Meyer-Olkin (KMO) Measure of Sampling Adequacy. The KMO index was 0.74, designating that the items were totally suitable for factor analysis (cut-off at 0.50). Furthermore, the Bartlett's Test of Sphericity was significant (*χ*^*2*^ (45) = 786.338, *p* < .001), meaning that the correlation matrix of the items was significantly different than an identity matrix, i.e. a matrix filled with zeros.

#### 3.1.2 The consequentialist scale factors

To explore the factorial structure of CS in the Greek sample, all 10 items of the instrument were subjected to an EFA following principal axis factoring with varimax rotation implemented by SPSS (IBM Corp. Released 2017. IBM SPSS Statistics for Windows, Version 25.0. Armonk, NY: IBM Corp.). We evaluated what was the optimal number of factors that explained a substantial proportion of variation within the data by computing the eigenvalues, i.e. the variance in all variables that was accounted for by a given factor. Higher factor eigenvalues denote better explanation of the variances of the variables. According to the Kaiser's criterion of eigenvalues greater than 1, results yielded a 3-factor solution as the best fit for the data (eigenvalue>1; scree plot available on request from the authors), accounting for 56.23% of the variance. The results of the EFA are presented in Table 2. Factor 2 obtained had exactly the same structure as in the original study [21]. Factor 2 represents the Deontology dimension with five items (numbers 1–5). This factor had an eigenvalue of 2.76 and accounted for 27.59% of the variance. Interestingly, the original dimension of Consequentialism, however, split up in two different dimensions: items number 6–8 loaded on one factor, with an eigenvalue of 1.84, accounting for 18.41% of the variance, while items 9 and 10 loaded on another factor with an eigenvalue of 1.02 and accounted for 10.23% of the variance (see Table 2, Factors 1 and 3 respectively).

**Table 2:** Exploratory Factor Analysis of the Items of the Consequentialist Scale on the whole sample (*N* = 415).

Items	Factor		
	1	2	3
*Consequentialism1*			
7	**0.852**	−0.036	0.171
8	**0.667**	−0.096	0.265
6	**0.542**	−0.100	0.410
*Deontology*			
4	−0.082	**0.535**	−0.051
3	−0.001	**0.516**	−0.016
5	−0.144	**0.479**	−0.031
2	0.095	**0.440**	0.149
1	−0.305	**0.389**	0.040
*Consequentialism2*			
9	0.293	0.002	**0.657**
10	0.093	0.054	**0.514**
Cronbach's *α*			
	0.780	0.577	0.525

*Notes.* Extraction method: Principal Axis Factoring; Rotation method: Varimax with Kaiser Normalization. Loadings >0.4 are in bold. Total variance explained: 56.23%.

Reliability estimates of these factor scores were obtained by calculating the Cronbach's *α* reliability coefficients for the items defining each of the three factors. The Cronbach's *α* values were found to be good for the first factor but low for the second and third factor (Table 2). Table S1 in Supplementary material shows Cronbach's *α* when each item of a factor is deleted.

As the factor structure was not as expected based on the original paper [21], we examined potential effects of age. We thus conducted Pearson's *r* correlations between participants' responses to each item and age. Results revealed significant correlations between age and item 2 (*r* = 0.156, *p* = .001), item 3 (*r* = 0.178, *p* < .001), item 5 (*r* = 0.119, *p* = .015, uncorrected), and item 9 (*r* = −0.120, *p* = .014, uncorrected). There was no significant correlation between age and any of the rest of the items (1: *r* = −0.061, *p* = .217; 4: *r* = 0.067, *p* = .171; 6: *r* = 0.022, *p* = .656; 7: *r* = 0.046, *p* = .353; 8: *r* = 0.020, *p* = .689; 10: *r* = −0.079, *p* = .109). There was a significant positive correlation between age and deontology scores (Spearman's *rho* = 0.154, *p* = .002), but correlation between age and consequentialism was not significant (Spearman's *rho* = −0.055, *p* = .268). Independent *t*-tests showed no significant differences neither between the age of participants with vs. without studies on philosophy (*t* (362) = −0.776, *p* = .438) nor between the age of participants with vs. without engagement with philosophy (*t* (362) = 0.578, *p* = .564).

We thus performed the EFA separately for two age groups of participants. We did a median split on our initial sample, resulting in a younger group less or equal than 36 years old (*N* = 215, *M* ± *SD* 24.9 *±* 5.4) and an older group more than 36 years old (*N* = 198, *M* ± *SD* 47.0 *±* 7.5). With regard to the younger group, according to the Kaiser's criterion of eigenvalues and parallel analysis a 2-factor solution was the best fit for the data (eigenvalue>1; scree plot available on request from the authors), accounting for 45.19% of the variance. The results of the EFA are presented in Table 3. Both Factors had exactly the same structure as in the original study. Factor 1 represented the Consequentialism dimension with five items (item numbers 6–10) with an eigenvalue of 2.47 accounting for 24.74% of the variance. Factor 2 represented the Deontology dimension with five items (item numbers 1–5) with an eigenvalue of 2.05 accounting for 20.45% of the variance.

**Table 3:** Exploratory Factor Analysis of the Items of the Consequentialist Scale separately for the younger and older groups of participants.

Younger			Older			
Items per factor	Factor		Items per factor	Factor		
*Consequentialism*	1	2	*Consequentialism1*	1	2	3
6	**0.715**	−0.168	7	**0.832**	0.018	0.074
7	**0.660**	−0.206	8	**0.798**	−0.026	0.085
8	**0.609**	−0.175	6	**0.664**	−0.019	0.146
9	**0.508**	0.201	9	**0.613**	−0.073	0.341
10	0.359	0.225				
*Deontology*			*Deontology*			
4	0.049	**0.589**	5	−0.176	**0.597**	0.016
1	−0.142	**0.576**	3	0.018	**0.520**	0.006
3	−0.050	**0.471**	4	−0.179	**0.460**	−0.143
2	0.189	**0.458**	2	0.153	**0.449**	0.014
5	−0.134	0.362	1	−0.386	0.273	0.191
			*Consequentialism2*			
			10	0.206	−0.053	**0.831**
Cronbach's *α*						
	0.698	0.589		0.829	0.561	–

*Notes.* Extraction method: Principal Axis Factoring; Rotation method: Varimax with Kaiser Normalization. Loadings >0.4 are in bold. Total variance explained for the younger group was 45.19%, while for the older group 59.87%.

With regard to the older group, according to the Kaiser's criterion of eigenvalues and parallel analysis a 3-factor solution was the best fit for the data (eigenvalue>1; scree plot available on request from the authors), accounting for 59.87% of the variance. The results of the EFA are presented in Table 3. Factor 1 represents part of the original Consequentialism dimension with four items (item numbers 6–9). This factor had an eigenvalue of 3.10 and accounted for 31.03% of the variance. Factor 2 (i.e. Deontology, item numbers 1–5) had an eigenvalue of 1.76 and accounted for 17.59% of the variance. Item 10 (belonging to the Consequentialism dimension according to the original factor structure by Robinson) loaded on a third factor with an eigenvalue of 1.13 and accounted for 11.25% of the variance.

The Cronbach's *α* values were found to be good for the first factor but low for the second factor for both groups, and low for the third factor for the older group (see Table S1 in Supplementary material for Cronbach's *α* if item deleted). These findings suggest that in the Greek sample the younger group perceived the structure of the Consequentialist scale similar to Americans, while the older group differed from Americans.

#### 3.1.3 Hierarchical k-means clustering of participants

**Fig. 1:**
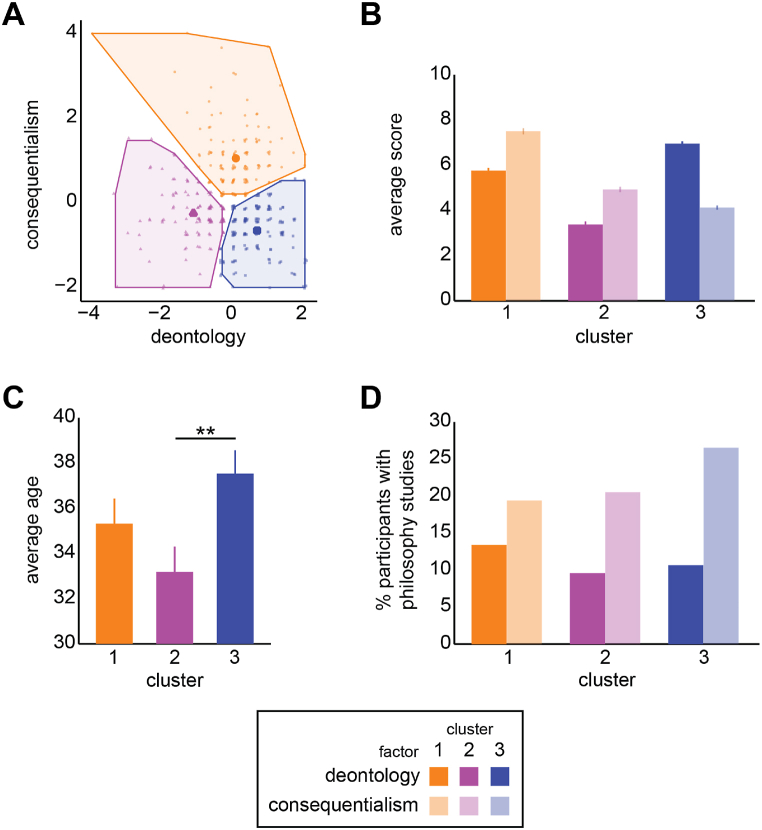
Results of the hierarchical k-means cluster analysis on participants based on their scores on the two factors of the Consequentialist scale, consequentialism and deontology, and effects of age and philosophy studies on cluster membership. **A** Cluster plot of the 415 participants from the cluster analysis (3 clusters: orange, purple, and blue). Participants are represented by points (jittered for visualization purposes). **B** Average factor scores (STEN) for each of the three clusters of participants, for each factor (consequentialism: opaque; deontology: transparent). **C** Average age for the three clusters of participants. **D** Percentage of participants with studies of philosophy. Error bars represent ±1 *SEM.* *p < .050, **p < .010. (For interpretation of the references to colour in this figure legend, the reader is referred to the Web version of this article.)

In order to investigate potential clusters of participants with similar patterns of responses in the deontology and consequentialism scales, we performed a hierarchical k-means clustering analysis based on the two factors. Specifically, the raw values (summed over each scale: items 1–5 for deontology, and items 6–10 for consequentialism) were z-scored and then converted to STEN scores, for simplicity and interpretability purposes. STEN scores are computed by multiplying the z-score with the standard deviation and adding the mean. The Deontology and Consequentialism scores (D and C scores) of each participant were subjected to hierarchical cluster analysis following Ward's method using squared Euclidean distances. The elbow method revealed that three was the most appropriate number of clusters (see Fig. S3). A non-hierarchical k-means algorithm using the same initial cluster centers improved the three-cluster solution (Fig. 1A). As shown in Fig. 1B, cluster 1 (*N* = 140) showed a medium D score and the highest C score, cluster 2 (*N* = 122) showed the lowest D score and a medium C score, while cluster 3 (*N* = 153) exhibited an opposite pattern, with the highest D score and the lowest C score. A 2 (factor: D, C) x 3 (cluster: 1, 2, 3) mixed ANOVA on the STEN scores revealed a significant factor × cluster interaction (*F* (2,412) = 234.833, *p* < .001, *η*^*2*^ = 0.533). All planned contrasts were significant at *p* < .001.

In order to investigate how age was distributed across the different clusters, we performed a one-way ANOVA with three levels (clusters) (Fig. 1C). There was a significant main effect of cluster (*F* (2,410) = 3.971, *p* = .020). Planned contrasts revealed that cluster 3 was comprised of significantly older participants (*M* = 37.53, *SD* = 12.69) compared to cluster 2 (*M* = 33.18, *SD* = 12.29) (*t* (272) = 2.854, *p* = .005), but there was no difference with cluster 1 (*M* = 35.31, *SD* = 13.11) (*p* > .1).

Furthermore, a chi-square test revealed a non-significant association between gender and cluster membership (*χ*^*2*^ (2, N = 410) = 2.438, *p* = .296) with cluster 1: 23.7% males (M) and 10.2% females (F), cluster 2: 18.3% M and 11.2% F, and cluster 3: 25.6% M and 11.0% F.

Finally, we examined potential contributions of previous experience with philosophy on cluster formation, by conducting chi-square tests between cluster membership and study of philosophy (Fig. XD) or engagement with philosophy. Results revealed non-significant associations between cluster membership and study of philosophy (*χ*^*2*^ (2, N = 366) = 4.448, *p* = .108) or study of philosophy (*χ*^*2*^ (2, N = 366) = 1.846, *p* = .397).

## 4 Discussion

### 4.1 Factor structure of the Consequentialist Scale in a native Greek-speaking sample - a culture effect hypothesis

Previous research has extensively studied moral ideologies in various populations, representing different professions, age groups, and cultural contexts. However, to the best of our knowledge, there are no studies of this kind in the Greek cultural context. In the present study, we examined the factor structure of the Consequentialist Scale (CS) in a sample of 415 adult Greek and Greek-Cypriot participants. We performed an Exploratory Factor Analysis (EFA) on the overall sample and found some deviations from the original factor structure proposed by Robinson [21]. While the original Deontology dimension with five items was replicated in its entirety, the original Consequentialist dimension did not fully align with the original structure, as it split into two separate factors.

A possible explanation for this discrepancy is the participants’ familiarity with the concepts of consequentialism and deontology. It is plausible that the participants in our study were more acquainted with the principles of deontology compared to consequentialism. This could indicate a cultural effect, as the consequentialist perspective is more prevalent in Anglo-Saxon and U.S. cultural contexts, while Greek culture tends to lean towards a deontological approach. Although there is no direct empirical evidence, findings related to cultural characteristics could indirectly support this view. For instance, studies have shown that societies characterized by higher relational mobility—the number of opportunities individuals have to form new relationships [41]—are more prevalent in Western Educated Industrialized Rich Democratic (WEIRD) societies [86,87]. Moreover, relational mobility has been linked to moral reasoning. Specifically, countries with low relational mobility tend to strongly reject utilitarian choices [43]. This negative association between relational mobility and utilitarianism—and by extension, consequentialism—can be further explained by the fact that societies with low relational mobility tend to prioritize perceived dignity [88], exhibit stronger social networks, adopt interdependent subsistence styles, and demonstrate higher adherence to social cues and norms [87,89]. These tendencies may promote more deontological moral reasoning (for a discussion on the possible reasons for the connection between relational mobility and rejection of utilitarian reasoning see [43]) and could indirectly explain our findings.

Cultural characteristics from Hofstede's typology could also contribute to a similar interpretation. For instance, uncertainty avoidance—the extent to which a culture tolerates ambiguity and uncertainty [42]—has been positively linked to a deontological commitment to duty [90]. It has also been positively associated with idealism and indirectly with deontology (to the extent that the absolutism sub-dimension of the idealism/relativism framework is related to deontology) and negatively with relativism [44,48]. Additionally, uncertainty avoidance has been positively linked to a tendency for formalization, task focus, and a psychological need for rules in both Greek [91] and non-Greek samples [92]. These findings align with studies reporting that uncertainty of outcomes and non-predictability of actions contribute to increased non-consequentialist decision-making and a greater reliance on deontological norms [93,94,95]. Given the above, the differentiation between the CS factors obtained by our sample and those reported by Robinson in his original study can be interpreted based on differences in the cultural characteristic of uncertainty avoidance. Greece presents a much higher level of uncertainty avoidance than the U.S [96,97]. Therefore, our Greek sample may demonstrate a greater understanding of and sensitivity to deontology compared to consequentialism. This differentiation could explain why only the composition of the deontology factor in the CS was fully replicated.

Nevertheless, other cultural characteristics from the same typology, like Individualism/Collectivism, seem to provide a more complex or even opposite picture. Greek culture, known for its collectivistic orientation, differs from the more individualistic American culture [96,42,98]. Some studies report a positive relationship between collectivism, idealism, and the attribution of importance to codes of behavior [46,48]. Additionally, a negative link has been found between collectivism and utilitarian moral reasoning [99,81]. These links could support an interpretation of our factor analysis where the Greek sample shows less familiarity with consequentialism compared to the American sample used by Robinson. However, other studies report a positive link between collectivism and utilitarian moral reasoning [100,101,102], suggesting the opposite relationship. Further adding to the complexity of the discussion, some studies report positive associations of collectivism with both deontology and consequentialism [45], as well as with both idealism and relativism [44].

In conclusion, it is evident that while some cultural characteristics such as relational mobility and uncertainty avoidance may provide partial explanations for the findings, the overall picture remains complex. To gain a clearer understanding, it would be beneficial to conduct studies that employ multiple instruments assessing various cultural characteristics in conjunction with a moral ideologies instrument like the CS. This would allow for an examination of interactions between these characteristics and a comparison of their relative influence on individuals’ moral ideologies. Moreover, for a valid cross-cultural comparison between Greeks and Americans, it is essential to administer these instruments to equivalent samples from both populations. However, to the best of our knowledge, no such comprehensive study has been conducted. Therefore, due to the absence of direct empirical data, further interpretations of the findings based on cultural effect hypotheses would be speculative and hypothetical.

Numerous other cultural characteristics could potentially offer indirect interpretations of our findings. Apart from relational mobility and Hofstede's typology, several other frameworks of such characteristics have been proposed (e.g. Refs. [103,104,105,106]). However, due to the limited space available in the current text, providing an extensive discussion of all the possible cultural factors that might have influenced our results is impossible. After all, when examining the factor loadings we obtained, it appears that explaining our data with a cultural context effect might not be entirely fitting. This is because the items of the reconstructed Deontology dimension have relatively low loadings, whereas the factor loadings of Factor 1 were good. As a result, the correlation among the original deontological items was not at the level expected to support the view that our participants were characterized by a better understanding of and higher familiarity with deontology. While the original deontological dimension was entirely reconstructed, it was not done with a high level of confidence from our participants. On the other hand, our study seems to reveal more nuanced findings concerning the effect of age.

### 4.2 Effects of age on the consequentialist scale

Numerous studies have revealed that age influences the propensity for certain moral ideologies [54,107,55,56,57,108,63,58,109,59,60,61], as well as the consistency and strictness of moral judgment [53,81,56,58,82,60]. Importantly, this age effect has been observed across cultures [54,79,59]. As mentioned earlier, a significant positive correlation between age and deontology scores was observed in our sample. Therefore, we decided to examine whether the factor divergence that we found in the overall sample would remain after an age-based split of our sample.

Interestingly, in our younger group of participants, the original factor structure was replicated entirely. In the older group, there was a divergence from the original factor structure. The Deontology factor was aligned with the original, but the Consequentialist factor was divided into two: one consisting of 4 out of 5 of the original factor loadings and the other with item 10. Considering that the factor structure arises through correlations among the scores of each item, this age effect could be explained by the fact that older people tend to hold more idealistic and deontological beliefs [53,54,55,56,57,58,59,60,61,62]. Moreover, it could be explained by the fact that older individuals tend to be stricter, more consistent, organized, and philosophically reflective in their moral thinking, with more developed but also fixed moral beliefs [53,80,81,56,58,82,60]. Therefore, they could be more sensitive to conceptual differences existing in statements regarding morality and more capable of detailed analyses of regulatory statements. The tendency of older people towards deontology and strictness of moral judgment can be explained by their proneness to negative affect [69,58] and empathetic involvement [64,68], as well as their need for regulation of emotional states [74,75,76], or even the salience of mortality in their minds [78,77]. These psychological mechanisms may have influenced the choices of the older participants in our study, leading to a high agreement and acceptance of the deontology items, resulting in a total reconstruction of the original deontology dimension of the CS, and a rather skeptical and hesitant reception of the consequentialist items, finally leading to a split of the original consequentialism dimension. However, we must stress that verifying such a psychological explanation in our sample would require the implementation of a new study assessing the aforementioned psychological mechanisms in addition to a moral ideologies questionnaire.

Notably, in our sample, the CS exhibited a good but relatively lower internal consistency for both the consequentialism (Cronbach's α = 0.780) and deontology (α = 0.58) dimensions compared to the original study by Robinson [21] (α = 0.80 and 0.69, respectively). Internal consistency was generally low in both age groups, except for consequentialism in the older group (α = 0.829), which was slightly higher than that of the original study. Interestingly, the consequentialist subscale consistently demonstrated higher internal consistency than deontology in other studies utilizing the CS in different cultural contexts [33,25,27]. This finding further weakens an interpretation of our results based on a cultural effect hypothesis, thereby supporting an interpretation primarily focused on the effect of age.

A cluster analysis conducted using the CS scores obtained from our native Greek-speaking sample provided additional insights into our results, particularly regarding the age effect. The analysis revealed the presence of three distinct clusters among the participants: Cluster 1 displayed a medium D score, the highest C score, and the highest overall scores for both dimensions; Cluster 2 had the lowest D score, a medium C score, and relatively low overall scores; Cluster 3 exhibited the highest D score and the lowest C score.

The higher scores in deontology compared to consequentialism observed in cluster 3 are particularly interesting, as this cluster primarily consists of significantly older participants. As mentioned above, previous studies have shown that older individuals tend to hold more idealistic and deontological beliefs [53,54,55,56,57,58,59,60,61,62]. This effect has been observed across cultures as well [54,59]. Although most of these studies utilize questionnaires related to the idealism/relativism framework of moral ideologies, they still support older people's inclination towards deontology, to the point that the absolutism sub-dimension of the idealism/relativism framework is closely related to deontology (for the relation between Forsyth's Ethics Position typology and the deontology/consequentialism framework, see [20]). Moreover, numerous studies employing moral dilemma vignettes have consistently reported that older individuals exhibit a propensity towards deontological moral judgment [65,66,58]. Importantly, this propensity has been observed across cultures [64,67]. In this sense, our findings regarding the age of participants in Cluster 3 align with the existing literature and provide further evidence of older people's inclination towards deontology.

Cluster 1, characterized by high D and C scores, consisted of participants with strong and unwavering moral beliefs. The lack of significant age differences between Cluster 3 and Cluster 1 can be explained by the aforementioned tendency of older people to exhibit consistency, organization, philosophical reflection, and strictness in their moral thinking [53,81,56,58,82,60], an effect observed across cultures [79]. Participants in Cluster 1 expressed strong and confident views in favor of both consequentialist and deontological statements, as evidenced by their high ratings. Given the findings from previous studies, it was expected that a significant portion of this group would consist of older individuals. Psychological mechanisms that are pronounced in older people, such as empathetic involvement in interpersonal communication and heightened negative affectivity, have been linked to strictness and consistency of their moral reasoning [64,58]. These mechanisms may have played a role in the formation of Cluster 1. However, it is important to mention again that validating such an explanation of our findings would require the design and implementation of a new study.

Cluster 2, characterized by balanced but relatively low D and C scores, may also align with the aforementioned empirical findings suggesting that older individuals exhibit more consistency, philosophical reflection, and strictness in their moral thinking, with well-developed and fixed moral beliefs [53,81,56,58,82,60], while younger individuals tend to be less consistent, more selfish, and more relativistic [55,56,57]. The equally low C and D scores in Cluster 2 might reflect that younger people reject both deontology and consequentialism due to their preference for more egoistic, selfish, or relativistic moral reasoning. Once again, our findings appear to be consistent with other studies that have reported an age effect on moral reasoning across different populations and cultures [54,79,59].

### 4.3 Other demographic variables

The results of our study revealed no significant effect of gender on the consequentialism scale. Empirical findings regarding gender effects on moral ideologies have been mixed. Some studies have reported a gender effect [107,110,108,109,111], while others have found no significant association [22,80,112,63,113]. However, most of the previous studies that found no effect of gender employed the idealism/relativism moral ideologies framework by Forsyth and Schlenker [3] using the Ethics Position Questionnaire. Additionally, studies based on the Deontology/Consequentialism framework have often used older questionnaires such as the SETA and the MEV. Therefore, further empirical research using more recent and refined deontology/consequentialism questionnaires is needed to investigate the potential gender effect in more detail.

Finally, we did not find any significant effects of experience or engagement with philosophy on the CS scale. Previous empirical studies have explored the association between moral ideologies and the level of education, with some reporting a significant relationship [109], while others found no effect [55,63]. However, these studies primarily focused on the Idealism/Relativism framework and examined the level of education without considering specific areas of expertise. To our knowledge, only a few empirical studies have investigated the potential effect of philosophy education at the university level or systematic amateur engagement with philosophy on moral ideologies [114]. Nevertheless, this is an area that warrants further focused empirical research, especially considering the evidence from studies demonstrating the effectiveness of ethics training programs on people's moral judgment and conduct [115,116,117,118,119,120].

## 5 Limitations

The main limitation of our study was that it only conducted an Exploratory Factor Analysis (EFA) of the CS. A comprehensive examination of the factor structure and validity of the CS in the native Greek-speaking context would ideally require a Confirmatory Factor Analysis (CFA). However, conducting a CFA would necessitate a new sample of participants, as our sample size only allowed for one of these two analyses to be performed. We chose to prioritize the EFA as it provides information about all possible factor loadings, including cross-loadings, for each item in every factor. On the other hand, CFA does not provide this detailed information as it constrain all factor loadings to zero, estimating only one loading per item [39]. Given the limitation in our sample size, the EFA was deemed more suitable for our investigation. By using EFA as a first step, we avoided biasing the results with prior expectations about the underlying factor structure.

Second, we did not assess the convergent validity of the CS by comparing it with another instrument that measures the propensity for Deontology/Consequentialism. This was because there were no validated questionnaires of this nature available in Greek. Further research is needed to examine the underlying causes of the discrepancies between the original factor structure and the one observed in our sample. The cultural effect hypothesis requires replication of the current findings on larger Greek samples and further investigation of the most prevailing moral ideologies in the Greek cultural context, as well as the possible links between these ideologies and several cultural characteristics like relational mobility, uncertainty avoidance, and others. Moreover, cross-cultural studies that utilize both the CS and instruments assessing these cultural characteristics in both Greek and non-Greek (e.g., U.S.) samples would be valuable. Given the limited existing research on moral cognition in Greek populations and the absence of cross-cultural studies comparing moral cognition between Greeks and Americans, our interpretation of the findings based on a cultural effect hypothesis had little empirical foundation. However, a more nuanced interpretation of our findings emerged from the observed age effect. When we divided our sample into older and younger participants, a clearer explanation for the discrepancies between the original factor structure and the one observed in our sample emerged. These discrepancies were only present in the group of older participants and were specific to the dimension of Consequentialism. This aligns with previous empirical research that consistently demonstrates older people's proneness toward stricter, more consistent, and more deontological moral reasoning. The clustering of scores within our sample further supports the age effect hypothesis, suggesting that the observed discrepancies may be attributed to the well-documented effect of age on the strictness and content of moral reasoning. Future research on moral cognition and moral ideologies in Greek samples will help expand upon the findings of the present study.

## 6 Conclusion

The aim of the present study was to validate the factor structure of the Consequentialist Scale (CS) in a sample of native Greek speakers, marking the first investigation of moral ideologies in a native Greek-speaking sample. Through Exploratory Factor Analysis (EFA), a three-factor solution emerged, deviating from the original two-factor structure that encompassed the dimensions of consequentialism and deontology. To investigate this further, separate EFAs were conducted for two age groups: younger and older participants. Interestingly, the younger group exhibited a two-factor solution that mirrored the original structure, while the older group displayed a three-factor solution. Additionally, a cluster analysis revealed that older participants formed a cluster with higher scores in deontology compared to consequentialism, whereas clusters comprising younger participants exhibited the reverse pattern. Although supporting a cultural effect hypothesis proved challenging, the age effect hypothesis provided a more nuanced interpretation of our findings, aligning with a substantial body of empirical research that explores the influence of age on moral reasoning. In this regard, our findings suggest that the well-documented and cross-culturally observed effect of age on the content and strictness of moral reasoning extends to Greek society. Gender and prior experience with philosophy did not significantly impact scores on the Consequentialist Scale. Overall, our findings indicate a satisfactory level of adaptation of the CS in the Greek cultural context.

## Funding statement

The study was funded by the Regional Governor of Attica-Athens-Greece, and co-funded by the Athanasios & Marina Martinou Foundation (AMMF) – non profit civil company AEGEAS.

## Author contribution statement

George Kosteletos: Conceived and designed the experiments; Performed the experiments; Analyzed and interpreted the data; Contributed reagents, materials, analysis tools or data; Wrote the paper.

Ioanna Zioga: Conceived and designed the experiments; Analyzed and interpreted the data; Contributed reagents, materials, analysis tools or data; Wrote the paper.

Evangelos D. Protopapadakis: Conceived and designed the experiments; Performed the experiments; Analyzed and interpreted the data; Contributed reagents, materials, analysis tools or data.

Andrie G. Panayiotou and Konstantinos Kontoangelos: Performed the experiments; Analyzed and interpreted the data; Contributed reagents, materials, analysis tools or data.

Charalabos Papageorgiou: Conceived and designed the experiments; Analyzed and interpreted the data.

## Data availability statement

Data will be made available on request.

## Declaration of competing interest

The authors declare that they have no known competing financial interests or personal relationships that could have appeared to influence the work reported in this paper.
