# Supplementary material for: The Consequentialist Scale: Translation and empirical investigation in a Greek sample
Source: Heliyon. 2023 Jul 17;9(7):e18386. doi: 10.1016/j.heliyon.2023.e18386 (PMC10393767; doi:10.1016/j.heliyon.2023.e18386)
Supplement: Multimedia component 1 [file mmc1.docx]

**Supplementary material**

**Inter-item correlation plot**


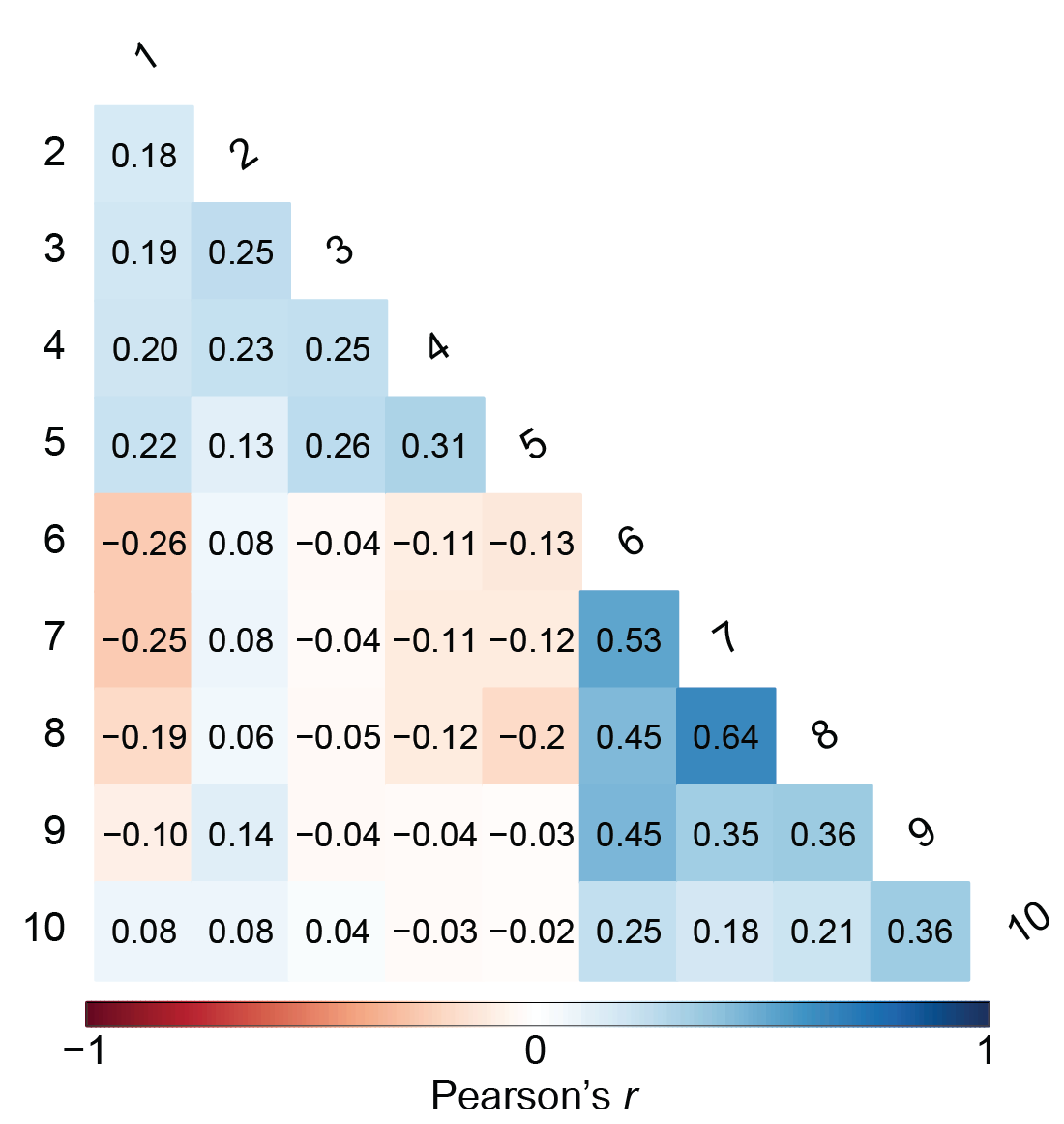


**Fig. S1** Pearson’s *r* correlation plot of participant’s responses between items 1-10 of the Consequentialist scale.

**Greek Translation of the Consequentialist Scale**

Παρακαλούμε υποδείξτε αν συμφωνείτε με τις ακόλουθες δηλώσεις. Κάθε μία εξ αυτών των δηλώσεων εκφράζει μια συνήθη κοινή πεποίθηση αλλά δεν υπάρχουν σωστές ή λανθασμένες απαντήσεις σε αυτές τις δηλώσεις. Μας ενδιαφέρει η δική σας απόκριση σε τέτοια ζητήματα γνώμης. Βαθμολογήστε την απάντησή σας σε κάθε μια από τις ακόλουθες δηλώσεις επιλέγοντας έναν αριθμό από το 1 ως το 5 όπου:

1 = διαφωνώ απόλυτα

2 = διαφωνώ

3 = ούτε συμφωνώ, ούτε διαφωνώ

4 = συμφωνώ

5 = συμφωνώ απόλυτα

1) Ορισμένοι κανόνες δεν πρέπει ποτέ να παραβιάζονται

**1**: διαφωνώ απόλυτα, **2**: διαφωνώ, **3**: ούτε συμφωνώ, ούτε διαφωνώ, **4**: συμφωνώ

**5**: συμφωνώ απόλυτα

2) Δε δικαιολογείται ποτέ ηθικά η πρόκληση βλάβης σε έναν άλλον άνθρωπο

**1**: διαφωνώ απόλυτα, **2**: διαφωνώ, **3**: ούτε συμφωνώ, ούτε διαφωνώ, **4**: συμφωνώ

**5**: συμφωνώ απόλυτα

3) Αν μια πράξη παραβιάζει τους βασικότερους κανόνες μιας κοινωνίας, δε θα πρέπει να λαμβάνει χώρα, ακόμα και αν έχει πολλά θετικά αποτελέσματα.

**1**: διαφωνώ απόλυτα, **2**: διαφωνώ, **3**: ούτε συμφωνώ, ούτε διαφωνώ, **4**: συμφωνώ

**5**: συμφωνώ απόλυτα

4) Ορισμένες πτυχές της ανθρωπότητας είναι ιερές και δεν πρέπει ποτέ να παραβιάζονται ανεξάρτητα από το όποιο πιθανό κέρδος.

**1**: διαφωνώ απόλυτα, **2**: διαφωνώ, **3**: ούτε συμφωνώ, ούτε διαφωνώ, **4**: συμφωνώ

**5**: συμφωνώ απόλυτα

5) Ορισμένοι κανόνες και νόμοι είναι οικουμενικοί και δεσμευτικοί ανεξάρτητα από τις περιστάσεις στις οποίες βρισκόμαστε.

**1**: διαφωνώ απόλυτα, **2**: διαφωνώ, **3**: ούτε συμφωνώ, ούτε διαφωνώ, **4**: συμφωνώ

**5**: συμφωνώ απόλυτα

6) Οι κανόνες και οι νόμοι είναι άσχετοι. Το μόνο που έχει σημασία όταν αποφασίζουμε πώς θα δράσουμε είναι το αν η δράση μας παράγει ευτυχία.

**1**: διαφωνώ απόλυτα, **2**: διαφωνώ, **3**: ούτε συμφωνώ, ούτε διαφωνώ, **4**: συμφωνώ

**5**: συμφωνώ απόλυτα

7) Οι κανόνες και οι νόμοι πρέπει να τηρούνται μόνο όταν μεγιστοποιούν την ευτυχία.

**1**: διαφωνώ απόλυτα, **2**: διαφωνώ, **3**: ούτε συμφωνώ, ούτε διαφωνώ, **4**: συμφωνώ

**5**: συμφωνώ απόλυτα

8) Εάν οι κανόνες και οι νόμοι δεν μεγιστοποιούν την ευτυχία για τους ανθρώπους, τότε θα πρέπει να αγνοούνται.

**1**: διαφωνώ απόλυτα, **2**: διαφωνώ, **3**: ούτε συμφωνώ, ούτε διαφωνώ, **4**: συμφωνώ

**5**: συμφωνώ απόλυτα

9) Η μόνη ηθική αρχή που πρέπει να ακολουθείται είναι η αρχή ότι πρέπει να μεγιστοποιούμε την ευτυχία.

**1**: διαφωνώ απόλυτα, **2**: διαφωνώ, **3**: ούτε συμφωνώ, ούτε διαφωνώ, **4**: συμφωνώ

**5**: συμφωνώ απόλυτα

10) Οι άνθρωποι που αποτυγχάνουν να μεγιστοποιήσουν την ευτυχία κάνουν κάτι ηθικά λάθος.

**1**: διαφωνώ απόλυτα, **2**: διαφωνώ, **3**: ούτε συμφωνώ, ούτε διαφωνώ, **4**: συμφωνώ

**5**: συμφωνώ απόλυτα

**Table S1.** Reliability analysis: Cronbach’s *α* for each factor and if item deleted, separately for all participants, the younger and the older group

| All participants | | Younger | | | Older | |
| --- | --- | --- | --- | --- | --- | --- |
| Cronbach’s *α* | Cronbach’s *α* if item deleted | Cronbach’s *α* | Cronbach’s *α* if item deleted | Cronbach’s *α* | | Cronbach’s *α* if item deleted |
| Conseq. 1 = .780 |  | Conseq. = .698 |  | Conseq. 1 = .829 | |  |
| 7 | 0.619 | 6 | .593 | 7 | | .751 |
| 8 | 0.690 | 7 | .623 | 8 | | .759 |
| 6 | 0.782 | 8 | .630 | 6 | | .803 |
|  |  | 9 | .671 | 9 | | .816 |
|  |  | 10 | .710 |  | |  |
|  |  |  |  |  | |  |
| Deont. = .577 |  | Deont. = .589 |  | Deont. = .561 | |  |
| 4 | 0.502 | 4 | .491 | 5 | | .443 |
| 3 | 0.498 | 1 | .502 | 3 | | .480 |
| 5 | 0.518 | 3 | .516 | 4 | | .509 |
| 2 | 0.554 | 2 | .578 | 2 | | .529 |
| 1 | 0.538 | 5 | .585 | 1 | | .561 |
|  |  |  |  |  | |  |
| Conseq. 2 = .525 |  |  |  | Conseq. 2 = - | |  |
| 9 | - |  |  | 10 | | - |
| 10 | - |  |  |  | |  |

**The Consequentialist Scale**

**Robinson, J. S. (2012). *The consequentialist scale: Elucidating the role of deontological and utilitarian beliefs in moral judgments* (Doctoral dissertation, University of Toronto).**

Please indicate if you agree or disagree with the following items. Each represents a commonly held opinion and there are no right or wrong answers. We are interested in your reaction to such matters of opinion. Rate your reaction to each statement by choosing a number from 1 to 5 where:

1 = completely disagree

2= disagree

3= neither agree or disagree

4= agree

5= completely agree

1) Some rules should never be broken

**1**: strongly disagree **2**: disagree **3**: neither agree or disagree **4**: agree **5**: strongly agree

2) It is never morally justified to cause someone harm.

**1**: strongly disagree **2**: disagree **3**: neither agree or disagree **4**: agree **5**: strongly agree

3) If an action is a violation of societies most basic rules it should not be committed; even if it will result in a large amount of good.

**1**: strongly disagree **2**: disagree **3**: neither agree or disagree **4**: agree **5**: strongly agree

4) Some aspects of humanity are sacred and should never be violated no matter the possible gain.

**1**: strongly disagree **2**: disagree **3**: neither agree or disagree **4**: agree **5**: strongly agree

5) Some rules and laws are universal and are binding no matter the circumstances you find yourself in.

**1**: strongly disagree **2**: disagree **3**: neither agree or disagree **4**: agree **5**: strongly agree

6) Rules and laws are irrelevant; whether an action produces happiness is all that matters when deciding how to act.

**1**: strongly disagree **2**: disagree **3**: neither agree or disagree **4**: agree **5**: strongly agree

7) Rules and laws should only be followed when they maximize happiness.

**1**: strongly disagree **2**: disagree **3**: neither agree or disagree **4**: agree **5**: strongly agree

8) If rules and laws do not maximize happiness for people they should be ignored.

**1**: strongly disagree **2**: disagree **3**: neither agree or disagree **4**: agree **5**: strongly agree

9) The only moral principle that needs to be followed is that one must maximize happiness.

**1**: strongly disagree **2**: disagree **3**: neither agree or disagree **4**: agree **5**: strongly agree

10) People that fail to maximize happiness are doing something morally wrong.

**1**: strongly disagree **2**: disagree **3**: neither agree or disagree **4**: agree **5**: strongly agree

**Demographic Questions**

1. Age
2. Gender
   1. male
   2. female
   3. other
3. Level of education
   1. primary school
   2. High school
   3. Lyceum
   4. University –first degree
   5. Master’s degree (Msc/MA)
   6. Phd
4. Have you studied anything related to Philosophy?
5. Do you have any -even amateur level- engagement related to Philosophy (e.g., reading of philosophical texts, audition of philosophical lectures/seminars, etc.)?
6. Profession
